# Carotenoids of Halophilic Archaea: Production, Properties and Biotechnological Applications

**DOI:** 10.1007/s12602-026-11076-w

**Published:** 2026-05-26

**Authors:** Pardeep Sheokand, Manoj Kumar Yadav, Bijender Singh, Svetoslav Dimitrov Todorov, Michael L. Chikindas, Santosh Kumar Tiwari

**Affiliations:** 1 Department of Genetics, Maharshi Dayanand University, Rohtak, 124001 Haryana India; 2 Department of Zoology, Rao Pahlad Singh Degree College, Balana, Mahendergarh, 123029 Haryana India; 3 Laboratory of Bioprocess Technology, Department of Microbiology, Maharshi Dayanand University, Rohtak, 124001 Haryana India; 4 Department of Biotechnology, Central University of Haryana, Mahendergarh, 123031 Haryana India; 5 ProBacLab, Laboratório de Microbiologia de Alimentos, Departamento de Alimentos E Nutrição Experimental, Food Research Center, Faculdade de Ciências Farmacêuticas, Universidade de São Paulo, São Paulo, SP 05508-000 Brazil; 6 CISAS- Center for Research and Development in Agrifood Systems and Sustainability, Instituto Politécnico de Viana Do Castelo, 4900-347 Viana Do Castelo, Portugal; 7 Health Promoting Naturals Laboratory, School of Environmental and Biological Sciences, the State University of New Jersey, 24 Rutgers, New Brunswick, NJ 08901 USA; 8 Department of General Hygiene, Sechenov First Moscow State Medical University, Trubetskaya Str. 8, Bldg. 2, Moscow, 119991 Russia

**Keywords:** Haloarchaea, Carotenoids, Bacterioruberin, Antimicrobial, Antioxidant, Anticancer

## Abstract

Halophilic Archaea possess fascinating physiological characteristics with unique ecological significance. Among the carotenoids, bacterioruberin is one of the most studied which is produced by different halophilic Archaea with beneficial characteristics such as antioxidant, antimicrobial and anticancer activity. Their metabolites exhibit distinctive characteristics such as stability in extreme conditions, structural modifications and biological activities, which may be used to develop innovative products for different biotechnological applications. At the laboratory level, carotenoid production depends on several physical and chemical parameters, such as incubation temperature, pH, and medium composition, in particular the presence and concentration of NaCl, CH_3_COONa, and MgSO₄. Thus, it is still *terra incognita*, an area that provides opportunities for research in the search of new natural compounds with numerous potential applications. This is a narrative review of carotenoids produced by halophilic archaea with an emphasis on biosynthesis, extraction, safety and potential biotechnological applications.

## Introduction

Since the discovery and recognition of Archaea as a domain of living organisms in 1990 by Karl Woos and George Fox, they have been formally identified as potential biotechnological microbial factories due to their unique characteristics. As part of the domain of Archaea, halophilic archaea represent even more intriguing and diverse group of microorganisms adapted to hypersaline environments. Haloarchaea show unique metabolic and structural features adopted through evolutionary processes as response for survival under osmotic stress. Halophilic microorganisms have generated interest in biotechnological industries due to their ability to synthesize high value carotenoids. These carotenoids can exhibit strong antioxidants, photoprotective, and membrane-stabilizing properties, thereby creating new opportunities for exploring halophilic archaea as potential candidates for production of pigments. Moreover, Archaea specificity and natural capacity to generate bioactive compounds highlight their potential for applications in food, cosmetic, and pharmaceutical industries. Thus, they can be considered as novel unexplored bioresource of microbial biotechnology.

In general, halophilic Archaea, also referred as haloarchaea, are a class of prokaryotic microorganisms that belong to the domain Archaea classified as the phylum Euryarchaeota. One of their main features is the ability of haloarchaea to thrive in environments with extremely high salt concentrations (3–5 M NaCl), much higher than that of seawater [1]. Moreover, few species of haloarchaea are capable of surviving on salt crystals [2] and occur mostly in hypersaline habitats such as salty lakes, solar salterns, salt deposits, salted fermented foods, and animal hides [3]. Culture-independent analyses of the gastrointestinal environment, including metagenomic approaches, can results and allow the isolation of haloarchaeal DNA from the gastrointestinal tract of animals with high salt intake diet [4]. However, such approaches should be analyzed and discussed with caution, as the detection and analysis of present DNA does not necessarily indicate the presence of living microorganisms in the intestinal microbiota of humans and other animals. Thus, as more correct alternative will be to work with RNA, as RNA is more correct representative for presence of living organisms. Haloarchaea microorganisms are also capable of growing under conditions of extreme hydrostatic pressure, low atmospheric pressure as that recorded on high altitudes, and in extremely hot and/or cold environments due to their metabolic specificity [5]. They are a significant source of various bioactive metabolites with potential for biotechnological and pharmaceutical applications. Evolutionary haloarchaea cells have adopted the "salt-in-cytoplasm" strategy to deal with osmotic stress in hypersaline environments, in which sodium chloride and/or potassium chloride are accumulated intracellularly at levels equivalent to those seen in the extracellular environment. Haloarchaea absorb potassium ions, which helps them to adapt to the osmotic pressure caused by the high salinity of the external environment [6]. Therefore, haloadaptation strategies to extreme conditions are developed through evolutionary processes, which have resulted in these microorganisms not only exhibiting tolerance but also, to some degree, dependence on the specific characteristics of their environment [7, 8]. For example, cells of *Haloferax larsenii* HA1 and other strains grow optimally in 15% salt and lyse in media with lower or no salt concentrations [9], conditions that are normally lethal for common representatives from the domain of Bacteria.

Interestingly, haloarchaea are less reported than other domains, such as Bacteria and Eukaryotic organisms in terms of scientific discovery, industrial applications and public awareness. This can be perhaps explained by their relatively late discovery and taxonomic identification. Moreover, their specific growth conditions, several questions regarding their unique properties are still considered as *terra incognita* and merit more scientific attention. The first report on Archaea dates to the 1970 s, when they were initially classified as a separate group of prokaryotes. The groundbreaking work by Woese et al. [10] led to the discovery of these unique organisms. The authors used ribosomal RNA sequencing to show that Archaea were distinct from both Bacteria and Eukaryotes, leading to the establishment of the three-domain system of life [10]. One of the earliest known reports of Archaea involved in the degradation of xenobiotic compounds was reported in the 1990s. A strain of *Haloarcula*, isolated from a salt marsh in France, showed the ability to degrade various aromatic compounds like anthracene and phenanthrene [11]. However, their characteristics and ecological behavior suggest vast potential for a wide range of biotechnological applications. The halo-adaptation strategies make them significant producers of metabolites with unique structures and functions. There are several biomolecules produced by haloarchaea such as halocins, carotenoids, bacteriorhodopsins, polyhydroxy-alkenoate, and polyhydroxy-butyrate, which have potential applications in several sectors including food, cosmetics, and therapeutics [12, 13]. The commercially available haloarchaea compounds include bacteriorhodopsin, squalene, diether, tetraether, and lipids. [14]. Bacterioruberin is the most characterized carotenoid of haloarchaea [15] which is used in pharmaceuticals, cosmetics, food industry, and biotechnological processes [16].

More than a thousand naturally occurring carotenoids have been characterized for their structural diversity and stability in extreme conditions [17]. They usually absorb visible light and are classified into two main groups: carotenes and xanthophylls. The structure of carotenes is composed of carbon and hydrogen, while xanthophylls contain at least one oxygen atom in their hydrocarbon chain [18]. The typical structure of carotenoids consists of isoprenoid units connected with conjugated double bonds leading to the synthesis of several stereoisomers with diverse physicochemical properties such as structure, stability, solubility, and absorption of light [19]. Carotenes generally contain C_40_ hydrocarbon backbone in 8 isoprene units and are also changed by different oxygen-containing functional groups which convert carotenes into xanthophylls [20, 21]. The examples of C_40_ carotenoids are β-carotene and lycopene, and C_30_ are isoprenoids (squalenes), and vitamin MK-8a [22].

Evidence related to potential health importance of carotenoids and their function as precursors of vitamin A biosynthesis in humans and other mammals have been found. There are several microorganisms other than Archaea such as algae, bacteria, and yeasts, which are also capable of producing carotenoids [22, 23]. Humans and other animals cannot produce carotenoids, but they can convert them to vitamin A. Microalgae, vegetables, and fruits are the primary source of precursors for retinol production [24]. Despite a variety of natural and synthetic sources of carotenoids, only a few, such as astaxanthin and β-carotene are produced commercially using microbial bioprocesses [25]. Bacterioruberin and its derivatives, monoanhydrobacterioruberin, bisanhydrobacterioruberin, tetrahydrobisanhydrobacterioruberin, and 2-isopentenyl-3,4-dehydrorhodopin are the most prevalent in the majority of the haloarchaea species studied so far [26].

The antimicrobial activity of haloarchaea carotenoids [27] has been reported against several public health pathogens such as *Klebsiella pneumoniae*, *Pseudomonas aeruginosa*, *Escherichia coli*, and *Staphylococcus aureus* indicated their potential applications in the preservation of salted foods and potential applications as new drugs for the treatment of infectious diseases. In addition, the higher antioxidant potential derived from commercially available compounds has attracted wider researchers to explore such molecules for therapeutic applications.

The commercial utilization and biotechnological potential of carotenoids are limited due to higher production costs, which may be minimized mainly through optimization of culture conditions and/or using agricultural and industrial waste as a nutrient source [28, 29]. Thus, new sources of carotenoids with cost effective bioproduction are highly desirable. Despite several advantages of haloarchaea and the unique characteristics of their pigments, there is limited information available on potential use in biomedicine, biotechnology, and pharmacy. Although research has attempted to provide data on the different carotenoids produced by haloarchaeal strains with possible applications [5, 12, 14, 17, 19], there are still gaps on purification and safety aspects.

Thus, the carotenoids of haloarchaea have potential for industrial applications due to their unique stability and enhanced functions. Although, more intense research is required for host range and mode of action of antimicrobial activity against targets, its antioxidant and anticancer potential are encouraging. The growth conditions can be optimized for higher biomass and extraction of these carotenoids involves comparatively fewer and easier steps for the recovery of haloarchaea carotenoid compared to other microbial and plant sources. This facilitates the large scale production of carotenoids for industrial applications. Further research is required focusing on the synergistic effect of these molecules with antimicrobial peptides and other antimicrobials. In addition, biosafety aspects need to be carefully explored before understanding the applications of haloarchaea carotenoids. This paper reports on existing literature on biosynthesis, extraction, and applications of carotenoids with emphasis on purification strategies and biosafety aspects important for industrial applications.

### Biosynthesis and Production of Carotenoids in Archaea

**Fig. 1:**
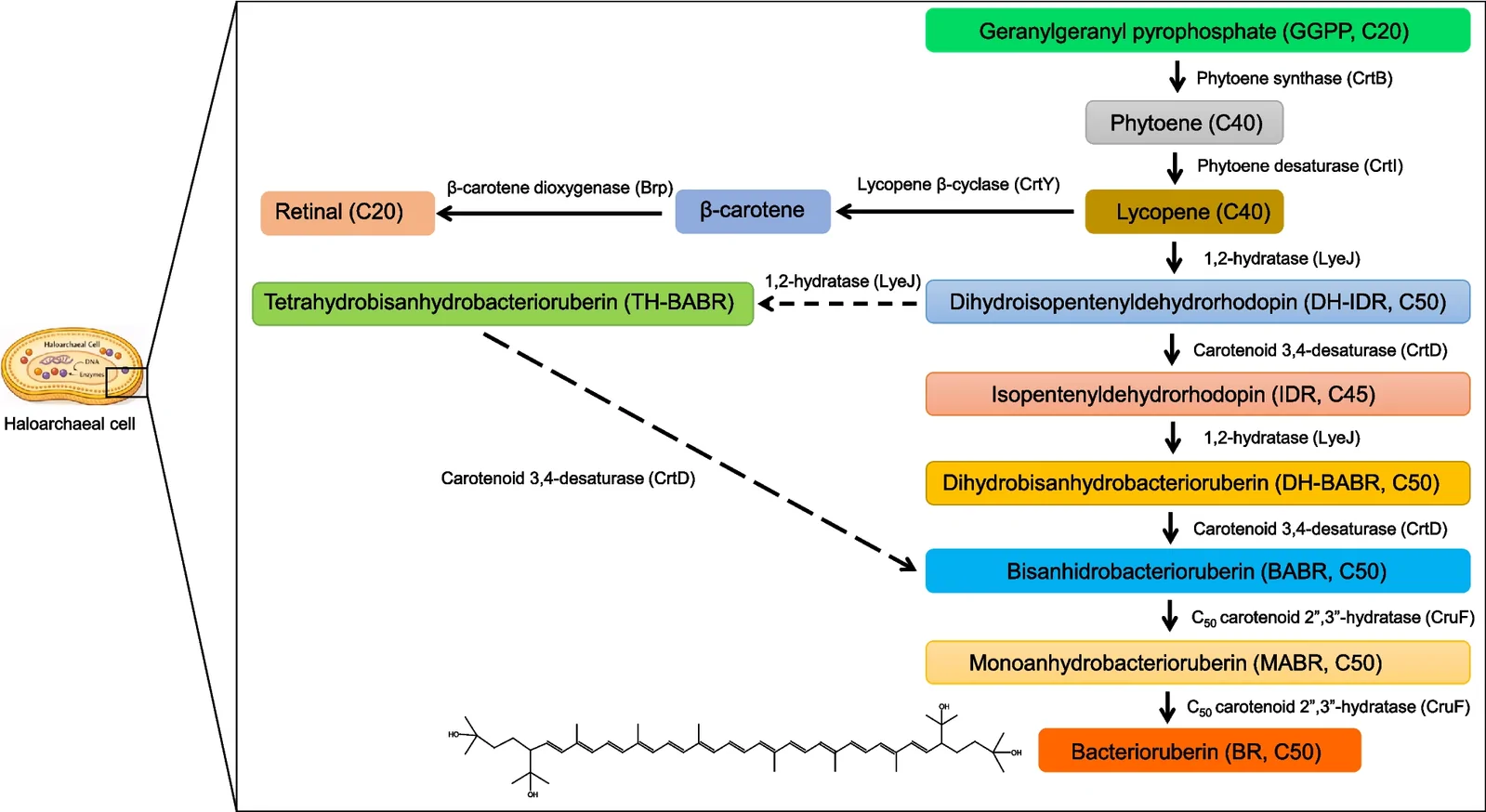
Biosynthesis of bacterioruberin, one of the most commonly synthesized carotenoid of haloarchaea

Biological production of carotenoids follows general metabolic pathways where the initial step of carotenoid biosynthesis is the condensation of two molecules of geranylgeranyl pyrophosphate (GGPP) to phytoene, a colorless precursor catalyzed by phytoene synthase[30, 31]. Further phytoene is subsequently converted to lycopene in the presence of phytoene desaturase [31, 32]. In organisms like anoxygenic photosynthetic bacteria, non-photosynthetic bacteria, and fungi phytoene desaturase are used to produce lycopene. However, oxygenic photosynthetic organisms (cyanobacteria, algae, and higher plants) convert phytoene to lycopene via carotene. Lycopene is further modified to produce a range of different carotenoids depending on haloarchaeal species. Few haloarchaea (e.g. *Halobacterium* spp. and *Haloferax* spp.) produce bacterioruberin, a red carotenoid pigment [31, 33, 34], and others (e.g. *Haloterrigena* spp.) produce β-carotene or lutein [33, 35]. In bacterioruberin biosynthetic pathway, lycopene is used as precursor and converted to bacterioruberin involving several intermediates and enzymes which are still not studied in detail. However, Yang et al. [36] have postulated that lycopene is first converted to dihydroisopentenyldehydrorhodopin (DH-IDR) followed by isopentenyldehydrorhodopin (IDR), dihydrobisanhydrobacterioruberin (DH-BABR), and bisanhidrobacterioruberin (BABR), which is further converted into monoanhydrobacterioruberin (MABR) by C_50_ carotenoid 2´, 3ˊ-hydratase. The same enzyme is responsible for the conversion of MABR into bacterioruberin [35]. In an alternate pathway, it is found that DH-IDR first converted to tetrahydrobisanhydrobacterioruberin (TH-BABR) and then BABR. Bacterioruberin is the main carotenoid synthesized by microorganisms belonging to the haloarchaea group and few species of *Micrococcus* 35]. The possible intermediates and enzymes involved in the biosynthesis of haloarchaeal carotenoids are shown in Fig. 1.

The presence of optimum conditions is a key parameter affecting the growth of haloarchaea and production of carotenoids. Several culture conditions such as salt, temperature, pH, carbon source, light, oxygen, and trace magnesium salts such as MgSO_4_, and MgCl_2_ affect the production of carotenoids. *Haloferax mediterranei* ATCC 33500 produces optimum level of carotenoids in the medium containing 15% NaCl. When these cells were grown in 20–35% NaCl, the production of carotenoid was 80 µg bacterioruberin per gram of cell proteins. *H. mediterranei* ATCC 33500 grown in a medium containing 15% NaCl produced 420 µg bacterioruberin per gram of cell proteins [37], whereas *Halobacterium salinarum* ATCC 33170 was unable to produce carotenoids at the same concentration [38]. *H. alexandrinus* TMT was able to grow in 25% NaCl with increased carotenoid production and there was no carotenoid production when the NaCl concentration was less than 10% [39].

Previous work reported that a medium containing 0.5% glucose resulted in higher production of bacterioruberin and β-carotene by *H. salinarum* ATCC 43214 and *H. salinarum* ATCC 33170, respectively [37]. The concentration of glycerol adversely affected the production of C_50_ red pigment while positively affecting the production of C_40_ carotenes. Moreover, it was generally suggested that glucose can stimulate microbial metabolisms, however, and glycerol can inhibit the conversion of C_40_ into C_50_ carotenoids.

Role of the light during the culturing of haloarchaea was investigated related to production of carotenoids. *H. salinarum* ATCC 33170 and *Haloferax alexandrinus* TM exhibited no colour change in the pigmentation when grown in the absence or presence of light [40]. It was reported that β-carotene and bacterioruberin were detected in *H. salinarum* JCM 10927 cells when cultured in the dark. Moreover, the production level of bacterioruberin increased up to three-folds, while the quantity of β-carotene declined in the presence of light. Bacterioruberin levels in *H. salinarum* JCM 10927 were reduced when cultured in the presence of light and at low oxygen levels, as hydroxylation requires oxygen. Total carotenoid concentration in dark-grown cells did not show changes significantly. Moreover, it was reported that low oxygen tension suppresses the production of bacterioruberin and increases the β-carotene or retinal concentration.

Trace magnesium salts such as MgSO_4_, and MgCl_2_ were suggested to affect the production of carotenoids. *H. alexandrinus* was cultured in a medium containing MgSO_4_.7H_2_O, MgCl_2_, or Na_2_SO_4_ to investigate role of magnesium and sulfate anions in relation to carotenoid production and cell development [39]. However, the carotenoid production and growth in haloarchaea were not validated in a medium containing less than 1% or more than 4% MgSO_4_. These findings indicate that the growth and carotenoid production by *H. alexandrinus* rely on the presence of magnesium and sulfate ions. The presence of magnesium may be attributed to facilitating cell division. Likewise, the demand for sulfate ions could be linked to their involvement in the cell wall composition of halophilic archaea.

MgSO_4_ is a common salt used in microbial culture media and plays an important role in providing microelements, supporting the physiology of both bacteria and archaea. Biological contribution of MgSO_4_ is primarily associated with providing the magnesium ion (Mg^2^⁺), a key cofactor required across all domains of life [41]. Magnesium as microelements, take part in numerous biochemical processes, including stabilization of ribosomal structure, activation of nucleic acid polymerases, and facilitation of ATP-dependent reactions. It is important to mention that ATP is biologically active only in its Mg-ATP complex was achieved and the viability of Mg^2^⁺essentially influences cellular energy metabolism. Moreover, magnesium plays essential role in the structural integrity of cellular membranes [42] by interacting with negatively charged phospholipids, thereby maintaining membrane fluidity and permeability under varying environmental conditions [43].

As part of the microbial culture media, MgSO_4_ serves as a direct source of soluble and bioavailable source of Mg^2^⁺. It is known fact that most of the bacteria exhibit strict requirements for magnesium, and even modest reductions in Mg^2^⁺ concentration can impair growth, disrupt cell division, and alter gene expression patterns. Beyond its role as a micronutrient, MgSO_4_ can influence microbial growth through its contribution to the osmotic environment and most probably in this regard its role in Archaea is key factor. Certain halotolerant and halophilic microorganisms, including both bacteria and archaea, can grow in environments dominated by aqueous MgSO_4_. In the evolutionary processes, these organisms have evolved specialized mechanisms to cope with the chaotropic and dehydrating effects of high-MgSO_4_ conditions, such as the accumulation of compatible solutes, modifications of membrane lipid composition, and the use of highly stable protein structures [44].

The sulfate ion (SO_4_^2^⁻) supplied by addition of MgSO_4_ to the growth media, plays a comparatively minor but still relevant role in microbial metabolism [45]. Many bacteria and archaea can assimilate sulfate as a sulfur source for the biosynthesis of amino acids such as cysteine and methionine, as well as to produce cofactors and coenzymes. However, in most laboratory media, sulfate is not the limiting sulfur component, and its contribution is overshadowed by the physiological importance of Mg^2^⁺. Different conditions required for the growth of haloarchaea, and the production of carotenoids are shown in Table 1.

**Table 1:** Culture conditions for the production of carotenoids by different species of haloarchaea

Haloarchaea	Optimum culture conditions	Carotenoids	References
*Natrialba* sp. M6	NaCl 25%, pH 10, 45 °C, 150 rpm	Nephthoside 1,2,3,4-tetraacetate, bacterioruberin, and monoanhydrobacterioruberin	[46]
*Haloferax mediterranei*	NaCl 12.03%, pH 8.96, 36.81 °C, 150 rpm	Bacterioruberin, monoanhydrobacterioruberin, and bisanhydrobacterioruberin	[47]
*H. mediterranei* ATCC 33500	NaCl 5%, MgSO_4_7H_2_O 8%, sodium acetate 0.1%, cultivation time 48 h	Bisanhydrobacterioruberin, monoanhydrobacterioruberin, bacterioruberin, and 2-isopentenyl-3,4-dehydrorhodopsin	[48]
*Halorubrum* sp. TBZ126	NaCl 20.55%, pH 7.94, 32 °C	Bacterioruberin, trans-lycopene, cis-lycopene, and trans-β-carotene	[49]
*Halorubrum* SP-4 *Halobacterium* SP-2	NaCl 15%, pH 8	Bacterioruberin	[50]
*H. alexandrines* JCM 10717	NaCl 25%, pH 7.2, 32 °C	Canthaxanthin, β-carotene, bacterioruberin, and astaxanthin	[39]
*H. alexandrines* TM (JCM 10717)	NaCl 10–25%, 32 °C, pH 7.2	Bacterioruberin, β-carotene, and canthaxanthin	[22]
*H. mediterranei* ATCC 33500	NaCl 5%, pH 7.5	Bacterioruberin	[51]
*H. mediterranei* ATCC 33500, *Halobacterium salinarum* ATCC 33170	NaCl 15%, pH 7.3, 38 °C	Squalene, dihydrosqualene, tetrahydrosqualene, β-carotene, and bacterioruberin	[52]

### Extraction and Purification of Carotenoids

**Fig. 2:**
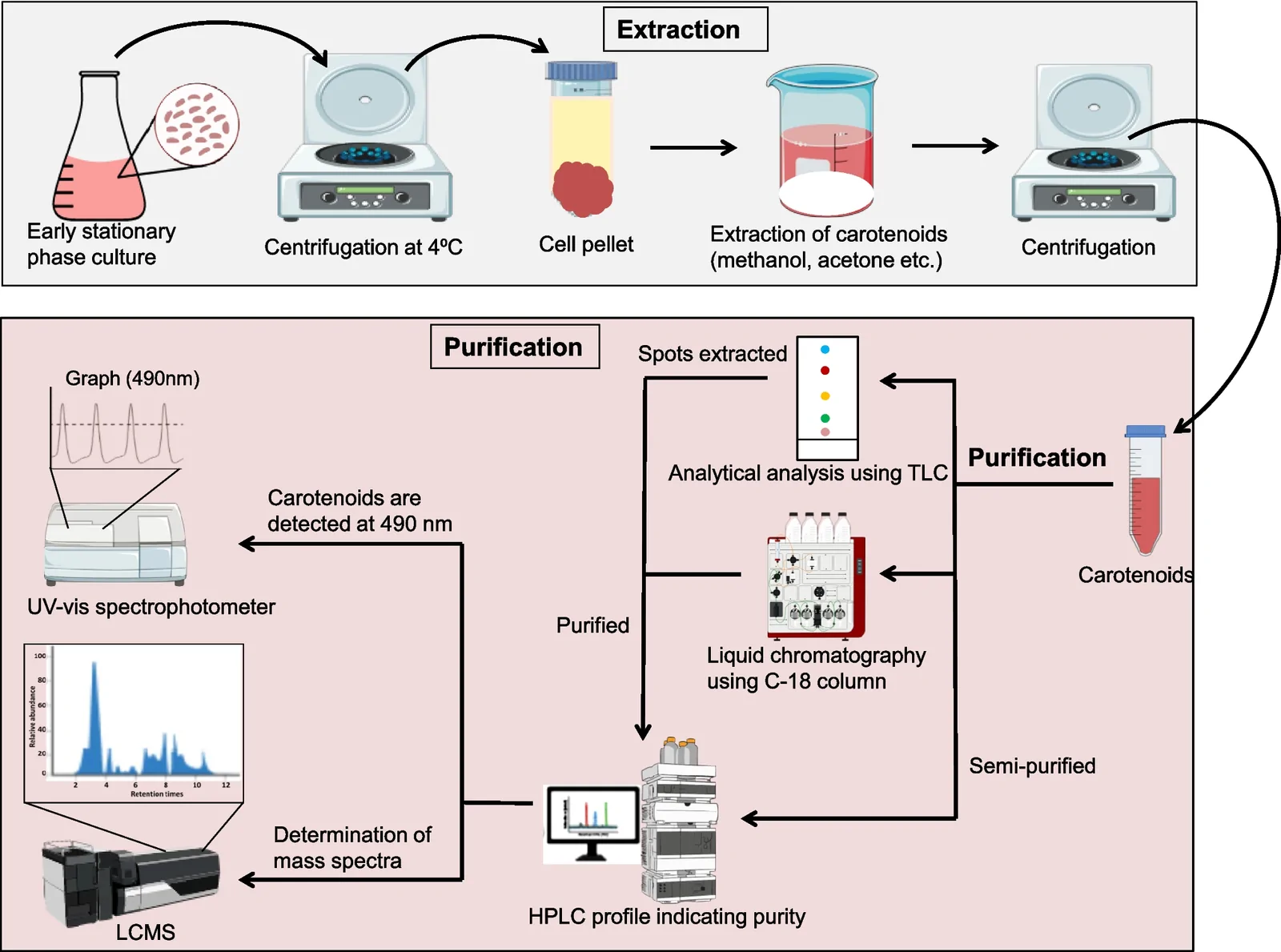
Different steps generally used during the extraction, purification and characterization of carotenoids of haloarchaea

Exploring beneficial properties and biotechnological applications of carotenoids by haloarchaea opens a need to develop effective extraction and purification strategies at a large scale for industrial applications. As carotenoids are membrane bound pigments, cells are generally harvested during the late log or beginning of the stationary phase of culture grown in the optimal conditions to get higher biomass and pigments. The cells are generally separated from spent medium using centrifugation at a low temperature (4 °C) and washed with saline to remove unwanted media components. The cells are suspended in suitable solvents such as methanol, acetone, hexane, etc. and further separated using centrifugation [53–55] (Fig. 2).

During the extraction, additional metabolites such as lipids, carbohydrates, proteins, and fatty acids are generally co-extracted along with carotenoids. Therefore, the separation of carotenoids to purity is essential in understanding their structure, function, and applications. However, for some industrial applications semi-purified or even crude extracts have also been used [14]. Lizama et al. [56] purified carotenoids from *Halorubrum tebenquichense* Te Se-85 using C-18 column with the mobile phase consisted of formic aqueous solution (1%), methanol formic acid (1%), and acetonitrile formic acid (1%). The purified carotenoid was detected using a diode array detector (DAD) at 490 nm as bacterioruberin and its derivatives, monoanhidrobacterioruberin and bisanhidrobacterioruberin [56]. Similarly, carotenoids from *Halorubrum* sp. SH1 were separated using high pressure liquid chromatography-DAD (HPLC–DAD) and ultra-high performance liquid chromatography–electrospray ionization tandem mass spectrometry (UHPLC-ESI–MS/MS) fitted with a C-18 analytical column with a binary mobile phase gradient consisted of acetonitrile:water:formic acid (84.9/15/0.1, *v/v/v*) in ethyl acetate. For the determination of mass spectra, liquid chromatography-mass spectroscopy equipped with electron spray ionization (LCMS-ESI) operating in positive scan mode (m/z 300–900) was used. The carotenoids detected in these cells were bacterioruberin, bisanhydrobacterioruberin, and trisanhydrobacterioruberin along with a few unidentified carotenoids [57]. Similarly, the carotenoids of *Halorubrum* sp. BS2 was also analyzed using an HPLC system equipped with a DAD and reverse-phase C-18 column and detected as bacterioruberin and bisanhydrobacterioruberin [27]. Carotenoids produced by *Halogeometricum rufum, H. limi, Haladaptatus litoreus, Haloplanus vescus, Halopelagius inordinatus, Halogranum rubrum,* and *Haloferax volcanii* were purified using a Shim-pack VP-ODS column and detected as bacterioruberin and its derivatives [33]. The carotenoids of *Haloarcula japonica* were extracted using HPLC fitted with a C-18 column. The elution was performed with 100% methanol and absorption spectra were recorded with a photodiode-array detector (250–580 nm). The phytoene and lycopene from *H. japonica* were identified using a combination of the HPLC retention times and the absorption spectra [58].

The carotenoid extract of *Haloterrigena thermotolerans* K15 was analyzed using thin-layer chromatography (TLC) with chloroform–methanol (93:7, *v/v*) as developing solvent and spots were examined in visible light. The spots were extracted from the TLC plate with methanol containing 0.2% butylated hydroxytoluene and analyzed using RP-HPLC with a photodiode array detector [59]. The spectra of the pigments of *Halobacterium* strain SP-2 were analyzed using a spectrophotometer and separated on a TLC silica gel plate with acetone and n-heptane (1:1, *v/v*) as the solvent system. The β-carotene was used as standard [48]. The pigments of *H. mediterranei* ATCC 33500 were also analyzed using TLC where silica was used as the solid phase, and 50% acetone in *n*-heptane (*v/v*) was as the development phase. In addition, the silica sorbent from each spot was scraped off, extracted with acetone, and scanned at 300–600 nm. Further analysis was performed using HPLC with a linear gradient consisting of 20% ethyl acetate in *n*-hexane and methanol, and elution was detected at 490 nm for the presence of carotenoids [48]. Thus, carotenoids of haloarchaea may be extracted from the cell biomass of freshly grown culture. The purification of specific carotenoids e.g. bacterioruberin may be achieved using C-18 column fitted with high-precision chromatography system. A combination of polar and non-polar solvents may be applied to separate the desired carotenoids from the mixture of extracts. Spectrophotometric detection is one of the reliable methods, but TLC is also used for the detection of carotenoids. A few important techniques used for the purification of haloarchaea carotenoids are mentioned in Table 2 and Fig. 2.

**Table 2:** Techniques used for purification and characterization carotenoids produced by different species of haloarchaea

Haloarchaea	Carotenoids	The technique used for analysis	References
*Natrialba* sp. M6	Nephthoside 1,2,3,4-tetraacetate, bacterioruberin and monoanhydrobacterioruberin	FTIR, Raman spectroscopy, GC–MS, LC–MS, and NMR	[46]
*Haloferax mediterranei*	Bacterioruberin, monoanhydrobacterioruberin and bisanhydrobacterioruberin	HPLC–MS	[47]
*H. mediterranei* ATCC 33500	Bisanhydrobacterioruberin, monoanhydrobacterioruberin, bacterioruberin and 2-isopentenyl-3,4-dehydrorhodopsin	TLC, and HPLC	[48]
*Halorubrum* sp. TBZ126	Bacterioruberin, trans- lycopene, cis- lycopene and trans- β-carotene	UV–visible spectrophotometer	[49]
*Halobacterium* SP-2*,* *Halobacterium* SP-4	Bacterioruberin	UV–visible spectrophotometer, TLC, and HPLC	[50]
*Halorubrum* sp. BS2	Bacterioruberin, monoanhydrobacterioruberin and bisanhydrobacterioruberin	HPLC, and LC/MS	[27]
*Halogeometricum* sp. ME3, *Haloarchula* sp. BT9, *Haloferax* sp. ME16	Bacterioruberin, monoanhydrobacterioruberin and bisanhydrobacterioruberin	HPLC, and LC/MS	[55]
*Haloplanus vescus* and *Halogeometricum limi*	Bacterioruberin, monoanhydrobacterioruberin and bisanhydrobacterioruberin	UV–visible spectrophotometer, TLC, and HPLC	[33]
*H alexandrines* JCM 10717	Canthaxanthin, β-carotene, bacterioruberin, and astaxanthin	HPLC	[39]
*H. alexandrines* strain JCM 10717	Bacterioruberin, β-carotene and canthaxanthin	HPLC	[22]
*H volcanii*	Bacterioruberin	HPLC, and GC–MS	[60]

### Characteristics of Carotenoids

The primary structure of carotenoids consists of an isoprenoid chain with a length of several carbon units conjugated with single and double bonds. Structure of bacterioruberin has a C-50 skeleton with thirteen conjugated double bonds and four hydroxyl groups [33]. It has two terminal groups which are linked with ether bonds to the polyene chain derived from isoprene units. Monoanhydrobacterioruberin has acyclic moiety at both termini and one ether bond between the two terminal groups [61]. Bisanhydrobacterioruberin also has two acyclic moieties instead of a hydroxyl group and has no ether bonds between the terminal cyclic groups and is the least polar of the three because these modifications reduce the polarity of the molecule and increase its lipophilicity [62]. The 2-isopentenyl-3,4-dehydrorhodopin, a C-45 carotenoid, consists of a linear chain of nine isoprene units with two hydroxyl groups at the ends and two double bonds in the middle. The presence of a 2-isopentenyl group at the 2-position is the characteristic feature of this carotenoid [61]. The dihydrobisanhydrobacterioruberin, a C-50 carotenoid, has two hydroxyl groups at C-1' and C-1'' of the terminal isoprene units, two double bonds at C-2' and C-2'' of the same units, and eight conjugated double bonds in the central polyene chain [35].

Carotenoids including bacterioruberin in halophilic species, integrate into archaeal membranes where their extended double-bond systems enhance rigidity and protect lipids from oxidative damage [61]. Functionally, archaeal carotenoids show higher antioxidant activity due to presence of a greater number of conjugated double bonds, stabilize membranes under extreme salinity or radiation, and contribute to coloration. The presence of carotenoids in the membrane acts as water barrier allowing ions and oxygen molecules to permeate through the cell membrane. Thus, these carotenoids can stabilize archaeal cells under high osmotic stresses. Their structural diversity and resilience support survival in harsh environments, highlighting their ecological and biotechnological importance [61].

Moreover, carotenoids play an important biological role in haloarchaea cells to protect them from several extreme conditions. It has been reported that bacterioruberin and its derivatives are responsible for the red/pink colour of the members belonging to *Halobacteriaceae* and *Haloferacaceae*. Bacterioruberin is an important antioxidant that protects the cells against oxidative damage. This property is due to the presence of 13 pairs of conjugative double bonds and makes haloarchaea resistant to radiation, strong light and hydrogen peroxide [63]. Due to presence of hydroxyl group, bacterioruberin increases membrane rigidity which decreases water permeability and increases the permeability of oxygen and other molecules leading to better survival of haloarchaea strains in cold and/or hypersaline environments [64]. It has also been reported that the rigidity of the cell membrane protects the haloarchaea from DNA-damaging agents [37]. The structural analysis has demonstrated that bacterioruberin is a part of the structure of archeorhodopsin which is a retinal protein-carotenoid complex and is involved in energy production in a few members of haloarchaea [63].

### Biotechnological Applications of Archaeal Carotenoids

Several metabolites of haloarchaea such as enzymes, carotenoids, halocins have potential applications in food, pharmaceuticals, and agriculture [13, 17, 31]. Carotenoids are one of the most commonly occurring compounds produced by different strains of haloarchaea which generally perform protective functions against extreme environments. In addition, it has been reported that such carotenoids, either crude extract or purified, have potential biotechnological applications [14, 17, 19]. A few applications of carotenoids have been described below and shown in Table 3.

**Table 3:** Biomedical applications of haloarchaea carotenoids

Carotenoids	Sources of carotenoids	Applications	References
Bacterioruberin, monoanhydrobacterioruberin and bisanhydrobacterioruberin	*Halobacterium halobium, Haloplanus vescus,* and *Halogeometricum limi*	Anticancer activity against human hepatoma cells	[33, 65]
Nephthoside 1,2,3,4-tetraacetate, bacterioruberin and monoanhydrobacterioruberin	*Natrialba* sp. M6	Induce apoptosis in Caco-2, HepG-2, and Hela cancer cell lines	[46]
Bacterioruberin	*H. halobium*	H_2_O_2_ and arachidonic acid protect in opposition to oxidative damage	[65]
Haloarchaeal extracts	*Halobacteria*	Decrease of tumor cells by radiation and combination treatment	[66]
Bacterioruberin, monoanhydrobacterioruberin and bisanhydrobacterioruberin	*Haloferax volcanii, Halogranum rubrum, Halopelagius inordinatus,* and *Halogeometricum rufum*	Protection against H_2_O_2_ in erythrocytes	[33]
Bacterioruberin, monoanhydrobacterioruberin and bisanhydrobacterioruberin	*H. volcanii, H. rubrum, H. inordinatus* and *H. rufum*	Scavenging activity	[33]
Bacterioruberin	*H. volcanii*	Improve cell viability, progressive motility, and sperm velocity	[60]
Bacterioruberin, monoanhydrobacterioruberin and bisanhydrobacterioruberin	*Halogeometricum* sp. ME3, *Haloarchula* sp. BT9, *Haloferax* sp. ME16, *Halorubrum* sp.BS2	Antimicrobial activity against human pathogens, *E. coli*, *Klebsiella pneumonia*, *Pseudomonas aeruginosa*, *Staphylococcus aureus,* and fish pathogens, *Aeromonas salmonicida*, *Vibrio anguillarum, Photobacterium damselae,* and *P. anguilliseptica*	[27, 55]
Nephthoside 1,2ˊ3ˊ4ˊ-tetraacetate, bacterioruberin and monoanhydrobacterioruberin	*Natrialba* sp. M6	Inhibitory potential against polymerase-dependent viral replication of hepatitis virus C and hepatitis virus B	[46]

#### Antioxidant Activity

Carotenoids demonstrate unique antioxidant activity due to presence of double bond structure and ability to delocalize unpaired electrons. This is the reason carotenoids quench free radicals such as hydroxyl (^•^OH), superoxide (O^•^_2_), and peroxyl (ROO^•^) radicals. The oxidation process in the cells involves the production of reactive nitrogen species (RNS) and reactive oxygen species (ROS) known as free radicals causing tissue injuries. Free radicals contain unpaired electrons produced as byproducts of metabolic reactions due to external stimulation [67]. When there is an imbalance production of ROS and RNS, it negatively affects the cellular structure by excessive oxidation or nitration of lipids, nucleic acids and proteins which is the beginning of many progressive diseases including diabetes, cancer and heart diseases [61].

Antioxidant compounds inhibit free radical reactions by interfering with oxidation process and preventing its damaging effects in plants and animals [68, 69]. There are several synthetic antioxidants such as tert-butylhydroquinone, butylated hydroxytoluene, butylated hydroxyanisole, and propyl gallate have been used in food and therapeutics but negatively impact the health, causing toxicity and DNA damage [70]. Therefore, there is a need for effective, non-toxic and natural antioxidants.

In this context, microorganisms like haloarchaea may be explored for the commercial production of carotenoids due to easy cultivation, growth manipulation and accessibility [54]. Bacterioruberin of haloarchaea generally shows higher antioxidant activity than the carotenoids from other organisms and the commercially available antioxidants. This could be due to the presence of a higher number of conjugated double bonds and OH groups in the haloarchaea carotenoids [71]. When compared to typical antioxidants, ascorbic acid, the carotenoid isolated from *Halorubrum* sp. BS2 shows higher antioxidant activity [27]. Similar findings have been reported from the carotenoids of *H. turkmenica, H. salinarum, H. volcanii, H. morrhuae,* and *H. rubrum*. The carotenoids of these strains have considerable and significantly stronger antioxidant activity than the standards such as β-carotene and ascorbic acid [33]. Thus, the carotenoids produced by haloarchaea are strong antioxidants with the capability to improve human health [63]. The antioxidant activity of crude carotenoid extracted from *Halorubrum* sp. SS12 was equivalent to the standard lutine and astaxanthin [72]. Several haloarchaeal genera namely *Halorubrum, Natronococcus, Natrialba, Haloferax, Halococcus, Halobacterium, Haloarcula,* and *Natrinema* were isolated from solar salt of Goa, India and identified as the producers of scavenges free radicles at varying levels. Bacterioruberin from *Haloferax* sp. GUSF-1 showed 2,2-diphenyl-1-picrylhydrazyl (DPPH) radical scavenging activity [73]. Alvare and Furtado [74] also studied the kinetic behavior of antioxidant activity of *H. alexandrines* GUSF1. Thus, several species of haloarchaea are reported to have higher antioxidant activity with potential therapeutic applications as compared to existing antioxidants. A comparison of antioxidant activity of carotenoids produced by different members of *Haloarchaea* with commercially available carotenoids has been depicted in Table 4.

**Table 4:** A comparison of antioxidant activity of carotenoids produced by different members of *Haloarchaea*

Carotenoid extracts from different haloarchaea	Antioxidant activity	Standard used	Antioxidant activity	Reference
*Halogeometricum* sp. ME3	IC50: 170.4	Ascorbic acid	IC50: 69.95	[55]
*Haloterrigena* sp. SGH1	IC50: 0.80	β-carotene	IC50: 4.20	[36]
*Halorubrum* sp. BS2	99%	Ascorbic acid	30%	[27]
*H. tebenquichense* TeSe-89	IC50: 23.19	Astaxanthin	IC50: 7.22	[75]
*H. tebenquichense* ALT 23	IC50: 8.83	Astaxanthin	IC50: 7.22	[36]
*Haloterrigenaturkmenica*	66.80%	Butylated hydroxytoluene	3.60%	[53]
*Haloterrigena thermotolerans* K15	36.87%	Ascorbic acid	14.89%	[59]
*Haloferax volcanii*	90%	β-carotene	14%	[33]
Bacterioruberin from genetically modified *H. volcanii* strain HVLON3	EC_50_ yielded 4.5 × 10^–5^ mol/l	Not available	Not availabl	[76]

The antioxidant activity was compared in terms of scavenging ability of 1,1-diphenyl-2-picrylhydrazyl radical (DPPH) for each carotenoid extract compared with respective standards using same concentration

#### Antimicrobial Activity

The rise of multidrug-resistant organisms and the failure of existing antibiotics have made the treatment of infectious illnesses challenging [77]. Thus, there is a need to explore alternative sources such as haloarchaea pigments for better efficacy and safety. Sahli et al. [27] reported the antibacterial activity of carotenoids from *Halorubrum* species BS2 against human pathogens such as *K. pneumoniae*, *P. aeruginosa*, *E. coli*, and *S. aureus.* The acetone extracts of *Haloarcula hispanica* HM1 and *H. salinarum* HM2 demonstrated effectiveness against bacteria, microalgae, and archaea, but not against yeasts. Antimicrobial activity varied among the targeted microorganisms such as *Micrococcus luteus, Vibrio harveyi, Dunaliella salina,* and *Halogeometricum* [78]. Hegazy et al. [46] reported the antiviral activity of carotenoids extracted from *Natrialba* sp. M6 against hepatitis C and hepatitis B viruses by inhibiting RNA polymerase and DNA-dependent DNA polymerase, respectively. The literature on antimicrobial potential of haloarchaeal carotenoids is limited only plate to assay and therefore, there is a further need to explore targeted host-range, exact mechanism of cell killing, resistance development and biosafety before their application in biomedicine.

#### Anticancer Activity

It has been reported that carotenoids from different haloarchaea strains were found to be effective against cancer cells. For example, anticancer potential of carotenoids of *H. limi* and *H. vescus* was studied in the liver hepatocellular carcinoma (HepG2) cell line [33]. Another example can be the haloalkaliphilic archaeon *Natrialba* species M6, demonstrating in vitro anticancer and apoptotic activity against colon, cervical, breast and liver cancer cell lines [46]. It was reported that carotenoids from *H. halobium* cause a reduction in HepG2 cell viability [65] mainly by inducing apoptosis, increasing gap junctional communication and arresting the cell cycle [79], whereas lycopene, a plant-based carotenoid was found to decrease HepG2 cell viability only at higher concentrations [80]. This indicated better efficacy of archaea-based carotenoids than plant carotenoids against cancer cells. Previous studies reported that bacterioruberin selectively inhibits the growth of breast cancer (MCF-7) and other cell lines. In addition, bacterioruberin induced caspase-mediated apoptosis better than the commercial agent, 5-fluorouracil which acts as suppressor of metalloprotease 9 [46], one of the key proteases involved in metastasis, angiogenesis and invasion [81]. The carotenoids extracted from *H. mediterranei* effectively decreased concentration-based cell viability of breast cancer cell lines [82]. Thus, it is apparent with the existing data that only in vitro studies are reported against cancer cell lines. The effect of different carotenoids was found to be cell line specific. There are very limited in vivo studies reported in animal models to the best of our knowledge. Therefore, there is urgent need for validation of in vitro studies in animals and humans to analyze the therapeutic applications for the treatment of cancer.

### Biosafety of Carotenoids

Carotenoids produced by biological sources are generally regarded as safe compared to synthetic compounds for applications in food, pharmaceutical, and cosmetic industries [37, 63]. It should be noted that there may be potential risks (e.g. impurities with unwanted side effects/toxicity) in the production of microbial products that need to be considered. When microbial products are used for human or other animal consumption, the manufacturing process should be designed to ensure that carotenoids are in pure form [83]. In some cases, although relatively rare, few people may experience allergic reactions to natural compounds, including carotenoids, and this must be considered and disclosed according to government regulations. Even though carotenoids are considered beneficial metabolites, excessive consumption may lead to carotenemia, a condition in which the skin turns yellow-orange, which may be harmless but can be mistaken for health complications. Additionally, although carotenoids from halophilic archaea are cited as having health benefits and several arguments are made in this regard, they must undergo rigorous testing to gain regulatory approval for use in consumer products to ensure their safety and effectiveness [37]. Appropriate research and risk assessment studies will need to consider the use of halophilic archaeal carotenoids in food, pharmaceutical products or supplements. Research on the use of these carotenoids for human health is still relatively sparse supporting the need before more research to understand biosafety evaluation and potential risks.

### Conclusions and Future Perspectives

**Fig. 3:**
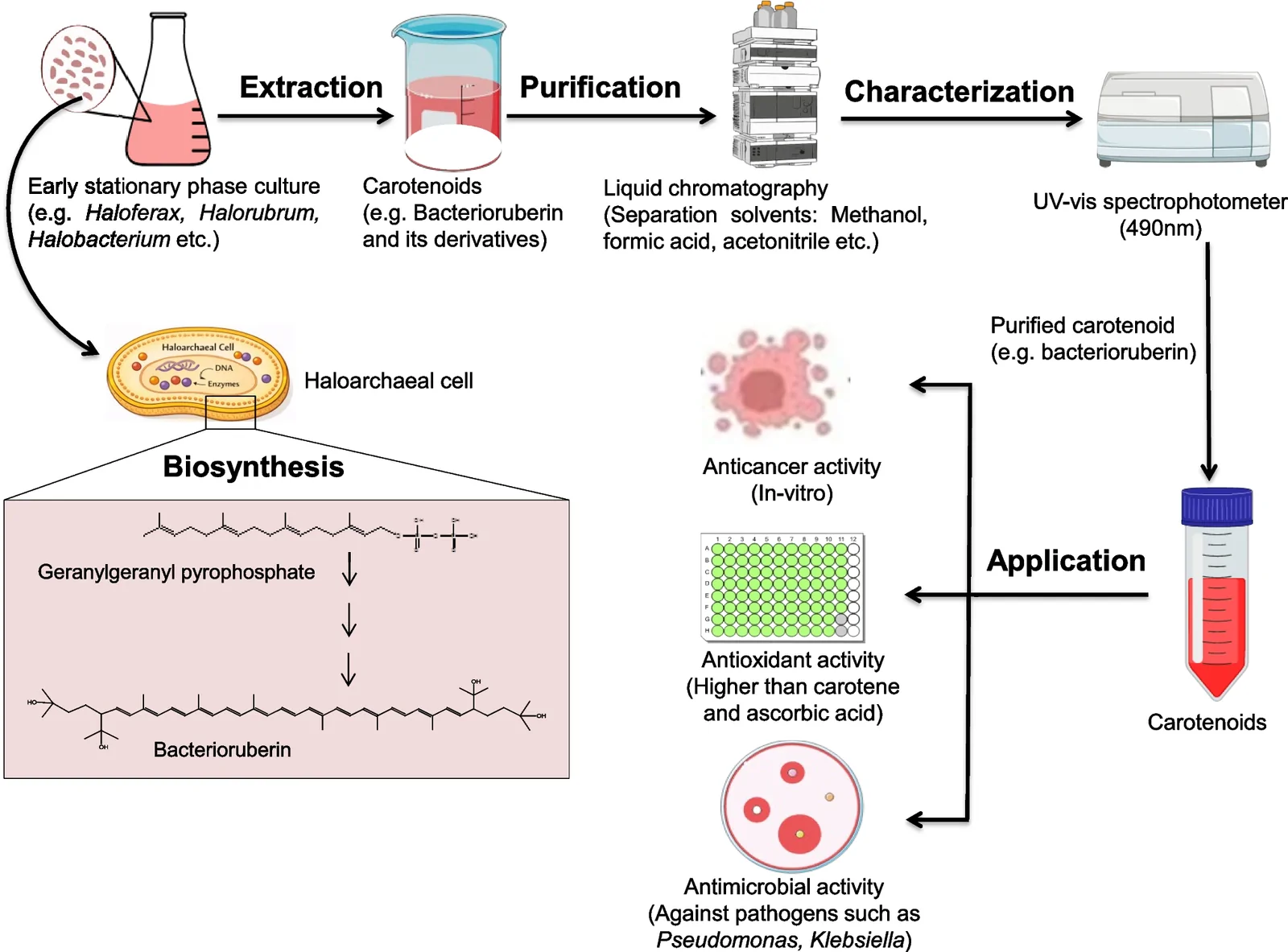
Biosynthesis, extraction and purification along with potential applications of carotenoids of haloarchaea

The ability of haloarchaea to adapt in extreme environments makes them suitable candidates to produce novel metabolites. Haloarchaea produce several compounds with unique features useful for industrial applications. These microorganisms may be potential choices for upscaling the production of carotenoids using cost-effective downstream processes. The extraction of carotenoids from haloarchaea involves reduce number of steps than plants and other sources making it comparatively economical for industrial production. The production of carotenoids in haloarchaea is mainly dependent on salt concentration; however, other components such as glucose and sodium acetate also influence the production of carotenoids. This review provides insights into the bioactive properties of carotenoids derived from haloarchaea for potential production of consumer products (Fig. 3). These compounds may have greater antioxidant potential than carotenoids from other microbes and plants. Improvement of carotenoid synthesis in strains with biotechnological potential can be achieved using modern approaches such as laboratory evolution and/or site-directed mutagenesis to manipulate genes and enzymes involved in the essential metabolic pathways (for review see Umeno et al. [84]). Furthermore, research on the molecular mechanisms underlying the unique adaptations of haloarchaea to extreme environments could lead to the development of novel carotenoids for industrial applications. In addition, the potential therapeutic applications of carotenoids such as their antioxidant, antimicrobial, and anticancer activities, justify further investigations for the development of novel drugs for public health.

## Data Availability

No datasets were generated or analysed during the current study.
